# The accuracy of haemoglobin A_2_ measurements in the presence and absence of haemoglobin S

**DOI:** 10.1002/jha2.691

**Published:** 2023-04-17

**Authors:** Jane K. Myburgh, Richard M. Szydlo, Barbara J. Bain

**Affiliations:** ^1^ Blood Sciences Imperial College Healthcare NHS Trust London UK; ^2^ Centre for Haematology Department of Immunology and Inflammation Hammersmith Hospital Campus of Imperial College Faculty of Medicine London UK; ^3^ Centre for Haematology Department of Immunology and Inflammation St Mary's Hospital Campus of Imperial College London London UK

**Keywords:** capillary electrophoresis, haemoglobin A_2_, high‐performance liquid chromatography, sickle cell anaemia, sickle cell trait, thalassaemia

## Abstract

The quantification of haemoglobin A_2_ by high‐performance liquid chromatography (HPLC) was compared with quantification by capillary electrophoresis for control subjects and patients with sickle cell trait or sickle cell anaemia. Significant differences were found, with estimated values being higher by HPLC for control subjects and higher by capillary electrophoresis for sickle cell trait and sickle cell anaemia patients. There is an ongoing need for improved standardisation and alignment of methods.

## 1 INTRODUCTION

The quantification of haemoglobin A_2_ is crucial in the diagnosis of β thalassaemia heterozygosity with small differences in the percentage being diagnostically important. However, problems have been noted with there being significant differences in measurements on different instruments, sometimes even with different instruments from the same manufacturer; in one study the mean values observed on nine instruments ranged from 2.2% to 2.8% [1]. In addition to problems with calibration and standardisation of methods, further complexity is introduced by the fact that high‐performance liquid chromatography (HPLC) separates what is termed haemoglobin A_0_ from two post‐translationally modified fractions, glycated and glutathionylated haemoglobin A. Any variant haemoglobin similarly has three fractions. This can result in the presence of haemoglobin S invalidating the A_2_ measurement because of post‐translationally modified haemoglobin S appearing in the same window as haemoglobin A_2_ [2]. This problem does not arise with capillary electrophoresis as the post‐translationally modified fractions do not separate from the main fraction [2, 3] To judge the clinical importance of this, we studied the haemoglobin A_2_ measurement on instruments based on two different principles in haematologically normal subjects and in patients with sickle cell anaemia and sickle cell heterozygosity (sickle cell trait).

## 2 METHODS

Estimates of haemoglobin A_2_ were made by HPLC and capillary electrophoresis, using a Bio‐Rad Variant II, Beta Thalassemia Short programme and Sebia Capillarys 3 Tera, respectively. Two Bio‐Rad variant II instruments were used on alternate days and each was therefore calibrated every two days. The Sebia Capillarys does not require calibration.

Testing was performed on specimens from 53 haematologically normal subjects and 50 patients with sickle cell trait and also on 149 specimens from patients with sickle cell anaemia, some of whom had been transfused. Specimens from patients with S/β thalassaemia and compound heterozygous states such as sickle cell/haemoglobin C disease were excluded. Analyses were performed on sickle cell trait and sickle cell anaemia samples over a 3‐month period in 2022 and analyses of normal samples in the subsequent 1‐month period. Comparisons of values for both the normal subjects and the sickle cell trait and sickle cell anaemia patients between the two types of the instrument were made using paired non‐parametric methods (Wilcoxon Signed Rank Test). The effect of the date of testing on differences between instruments was examined. Samples were in consecutive order by date so that the sample number could be used as a surrogate for the date of testing. For subject and patient samples (i.e. normal, sickle cell trait and sickle cell anaemia), values of the differences were divided into two equal groups (before and after the middle sample number). The differences were then compared using a t‐test.

## 3 RESULTS

**FIGURE 1:**
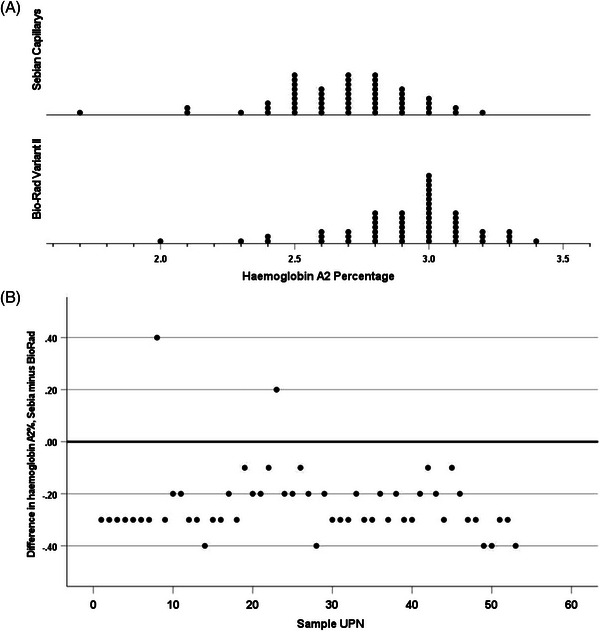
(A) Frequency plots of percentage haemoglobin A_2_ by high‐performance liquid chromatography (HPLC) and capillary electrophoresis in 53 normal subjects, median percentages being 3.0% and 2.7% respectively (*p* < 0.001); (B) Plot showing the difference between percentage haemoglobin A_2_ by HPLC and capillary electrophoresis in 53 normal subjects over time. The unique patient number (UPN) is plotted, these being in date order, drawn from a spreadsheet of all samples tested.

**FIGURE 2:**
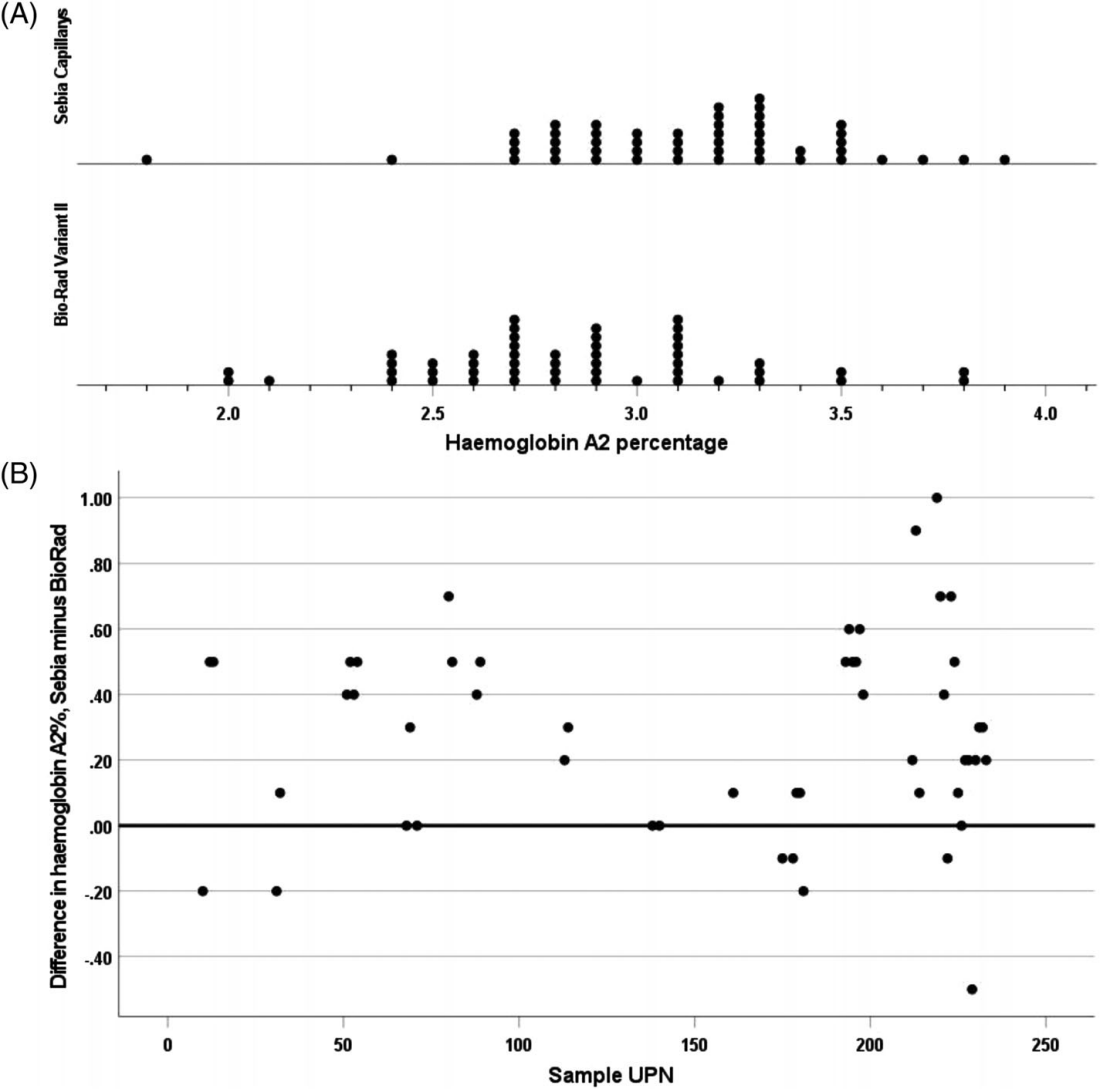
(A) Frequency plot of percentage haemoglobin A_2_ by high‐performance liquid chromatography (HPLC) and capillary electrophoresis in 50 subjects with sickle cell trait, median percentages being 2.8 and 3.2% respectively (*p* < 0.001); (B) Plot showing the difference between percentage haemoglobin A_2_ by HPLC and capillary electrophoresis in 50 subjects with sickle cell trait over time as shown by unique patient number (UPN).

**FIGURE 3:**
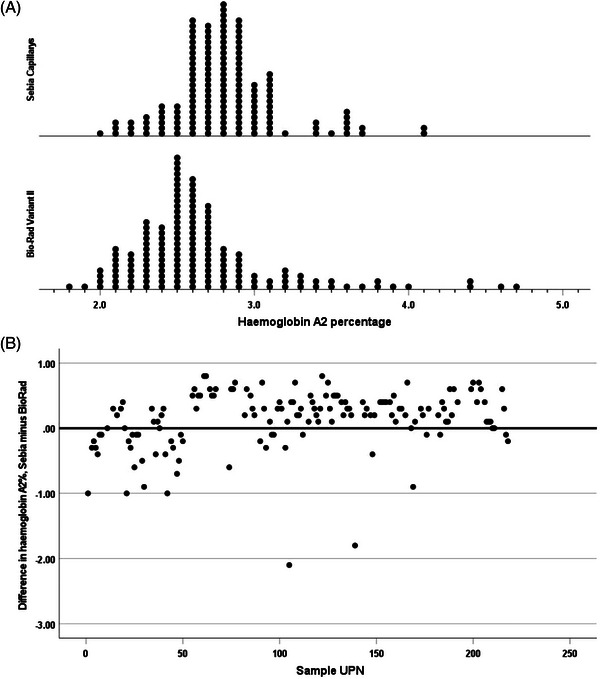
(A) Frequency plot of percentage haemoglobin A_2_ by high‐performance liquid chromatography (HPLC) and capillary electrophoresis in 149 subjects with sickle cell anaemia, median values being 2.6% and 2.8% respectively (*p* < 0.001); (B) Plot showing the difference between percentage haemoglobin A_2_ by HPLC and capillary electrophoresis in 149 subjects with sickle cell anaemia over time as shown by unique patient number (UPN).

Results of haemoglobin A_2_ estimation on normal subjects (*N* = 53) gave median values of 3.0% (range 2.0–3.4) by HPLC and 2.7% (range 1.7–3.2) by capillary electrophoresis. The median difference between the measurements was −0.3 (range −0.4–0.4) (*p* < 0.001). (Figure 1). For subjects with sickle cell trait (*N* = 50), the median values were 2.8% (range 2.0–3.8) by HPLC, and 3.2% (range 1.8–3.9) by capillary electrophoresis. The median difference between the measurements was 0.3 (range −0.5–1.0) (*p* < 0.001). We have previously established reference ranges for both methodologies, based on results in 78 haematologically normal subjects – for HPLC 2.34–3.2 (mean 2.77) and for capillary electrophoresis 2.25–3.05 (mean 2.65); [3] with both techniques there were a significant number of results in patients with sickle cell trait (4 and 7, respectively) falling above the upper limit of normal that had been established in this earlier study (Figure 2). For subjects with sickle cell anaemia (*N* = 149), the median values by HPLC were 2.6% (range 1.8–4.7), and 2.8% (range 2–4.1) by capillary electrophoresis. The median difference between the measurements was 0.2 (−2.1–0.8) (*p* < 0.001). However, on HPLC 17 of 149 specimens had values above the normal range, ranging from 3.3% to 4.7% while on capillary electrophoresis 26 specimens had values above 3.05% but ranging only up to 4.1% (Figure 3). For the sickle cell anaemia patient samples, the difference between the results from the two types of instruments altered over time (Figure 3B) (*p* 0.002). For the sickle cell trait samples, there was no significant change with time (*p* = 0.20). There was similarly no change over time for the normal samples (*p* = 0.091).

## 4 DISCUSSION

The diagnosis of β thalassaemia heterozygosity requires that quantification of haemoglobin A_2_ is both precise (reproducible) and accurate (close to ‘truth’). The difference in mean values with different technologies that have previously been reported [1] and the difference in median values of normal samples observed in this study indicate that accuracy has not yet been achieved despite attempts at standardisation.

We have observed capillary electrophoresis on normal samples to give lower haemoglobin A_2_ measurements than HPLC. This reflects our earlier results when we found A_2_ by capillary electrophoresis to have a median of 2.65% in normal subjects while HPLC had a median of 2.77% (*p* < 0.001). It also reflects the results observed by others for samples with a normal A_2_ including a comparison of BioRad Variant II HPLC with Sebia Capillarys 2 capillary electrophoresis [4] and a recent study using three capillary electrophoresis methods and five HPLC methods [1]. However interestingly the first of these two studies found a lower haemoglobin A_2_ by capillary electrophoresis when the haemoglobin A_2_ was elevated as a result of β thalassaemia heterozygosity while the latter found no difference between these two technologies. A further large study found that unselected diagnostic specimens measured in parallel on two instruments showed significantly lower haemoglobin A_2_ by capillary electrophoresis than by HPLC; the published figures also show that more high A_2_ values (above 3.5%) were observed by HPLC than by capillary electrophoresis [5]. A further investigation reported in the same paper in which the specimens from the same selected individuals were analysed for three consecutive years found capillary electrophoresis results to be lower in 2017 and 2018 but not in 2019, suggesting possible improvements in methodology [5].

We observed HPLC estimates of haemoglobin A_2_ to be higher than capillary electrophoresis estimates in normal subjects whereas they were lower in both sickle cell trait and sickle cell anaemia. Higgins et al. [4], however, found a significant difference only in non‐transfused subjects with sickle cell anaemia. In addition to differences between methodologies despite regular calibration, we observed a change in the relationship between the two technologies over time in sickle cell anaemia. This was not observed with normal subjects or in patients with sickle cell trait.

Elevated estimates of haemoglobin A_2_ in the presence of haemoglobin S were observed with HPLC but this was also seen with capillary electrophoresis. We had previously established reference ranges for these two methodologies based on 78 haematologically normal subjects, with capillary electrophoresis having a range of 2.25%–3.05% (mean 2.65) and HPLC having a range of 2.34%–3.2% (mean 2.77) [3]. In comparison with these reference ranges, four of 50 sickle cell trait patients had an elevated haemoglobin A_2_ by HPLC and seven of 50 by capillary electrophoresis. Of the patients with sickle cell anaemia, 17 of 149 had an elevated A_2_ by HPLC and 26/149 by capillary electrophoresis.

Our results are to some extent unexpected. On the basis of previous studies, we expected to find a factitious elevation of the haemoglobin A_2_ in the presence of haemoglobin S when HPLC was the method used. However, the increase in haemoglobin A_2_ on capillary electrophoresis in patients with haemoglobin S is unexpected and is yet to be explained. These results indicate the need for improved standardisation and great care in the interpretation of apparently elevated values in these patients. In addition, it should be noted that the findings relate to an HPLC instrument from a single manufacturer and results from other manufacturers’ instruments may well differ. It may be unwise to report the haemoglobin A_2_ percentage in the presence of haemoglobin S since results may not be valid and when reported to be elevated may cause confusion to clinical staff. Those reporting results need an in‐depth knowledge of the subject.

Overall, our results indicate that there is an ongoing need for standardisation and alignment of methods and reagents and for the calibration of instruments using an internationally agreed, certified reference material in order to achieve accurate results for the measurement of haemoglobin A_2_. The International Federation of Clinical Chemistry and Laboratory Medicine and the International Council for Standardization in Haematology continue their work to achieve this[5]. Reaching this objective will be important for the accurate diagnosis of β thalassaemia heterozygosity. Whether different reference ranges for HPLC and capillary electrophoresis are required needs to be established.

## AUTHOR CONTRIBUTIONS

Jane Myburgh and Barbara Bain collected the data. Richard Szydlo performed the statistical analysis. All authors contributed to the interpretation of the results.

## CONFLICT OF INTERESTS STATEMENT

The authors declare no conflict of interest.

## FUNDING INFORMATION

No funding was received.

## ETHICS STATEMENT

The authors have confirmed ethical approval statement is not needed for this submission.

## PATIENT CONSENT

The authors have confirmed patient consent statement is not needed for this submission.

## CLINICAL TRIAL REGISTRATION

The authors have confirmed clinical trial registration is not needed for this submission.

## Data Availability

Data are available from the authors at the request.
